# Coherent pathway enrichment estimation by modeling inter-pathway dependencies using regularized regression

**DOI:** 10.1093/bioinformatics/btad522

**Published:** 2023-08-23

**Authors:** Kim Philipp Jablonski, Niko Beerenwinkel

**Affiliations:** Department of Biosystems Science and Engineering, ETH Zurich, Basel 4058, Switzerland; SIB Swiss Institute of Bioinformatics, Basel 4058, Switzerland; Department of Biosystems Science and Engineering, ETH Zurich, Basel 4058, Switzerland; SIB Swiss Institute of Bioinformatics, Basel 4058, Switzerland

## Abstract

**Motivation:**

Gene set enrichment methods are a common tool to improve the interpretability of gene lists as obtained, for example, from differential gene expression analyses. They are based on computing whether dysregulated genes are located in certain biological pathways more often than expected by chance. Gene set enrichment tools rely on pre-existing pathway databases such as KEGG, Reactome, or the Gene Ontology. These databases are increasing in size and in the number of redundancies between pathways, which complicates the statistical enrichment computation.

**Results:**

We address this problem and develop a novel gene set enrichment method, called *pareg*, which is based on a regularized generalized linear model and directly incorporates dependencies between gene sets related to certain biological functions, for example, due to shared genes, in the enrichment computation. We show that *pareg* is more robust to noise than competing methods. Additionally, we demonstrate the ability of our method to recover known pathways as well as to suggest novel treatment targets in an exploratory analysis using breast cancer samples from TCGA.

**Availability and implementation:**

*pareg* is freely available as an R package on Bioconductor (https://bioconductor.org/packages/release/bioc/html/pareg.html) as well as on https://github.com/cbg-ethz/pareg. The GitHub repository also contains the Snakemake workflows needed to reproduce all results presented here.

## 1 Introduction

The behavior of cells is governed by a complex interplay of molecules. Their functional dynamics are organized according to biological pathways (Chuang *et al.* 2010). Perturbations of pathways have been linked to certain diseases, such as cancer (Hanahan and Weinberg 2000, 2011). Biological pathways can be obtained from pathway databases such as the Kyoto Encyclopedia of Genes and Genomes (KEGG), the Gene Ontology (GO), or Reactome (Ogata *et al.* 1999, Gene Ontology Consortium 2004, Joshi-Tope *et al.* 2005). It is important to note that pathways typically impose a structure of interactions in the form of a network on its contained molecules. While the nodes of this network typically correspond to genes, the edges correspond to interactions, such as signal transductions (Steffen *et al.* 2002). Another way of grouping genes in a meaningful way is to forgo the structure requirement and simply consider, for example, functionally related genes to be part of the same gene set.

Experiments investigating, for instance, differentially expressed genes between several conditions (e.g. wild-type versus mutant cell cultures) often produce a long list of genes of interest which is difficult to interpret (Simillion *et al.* 2017, Maleki *et al.* 2020). A common method for aggregating these lists of potentially interesting genes is to assess whether the genes preferentially appear in biologically relevant pathways. This reduces the amount of information which needs to be interpreted from individual genes to groups of genes, i.e. pathways, following a similar function.

There are several approaches to computing whether certain genes preferentially appear in certain gene sets. They can be roughly divided into three groups: (i) singular enrichment analysis, (ii) gene set enrichment analysis, and (iii) modular enrichment analysis (Huang *et al.* 2009). In a singular enrichment analysis, a list of genes resulting from a differential expression analysis is first partitioned into differentially expressed and not differentially expressed genes based on a threshold typically applied to effect size or *P*-value. These two groups of genes are then used to compute a pathway enrichment score individually. The gene set enrichment analysis lifts the requirement of a pre-selection of genes and considers all input genes without partitioning them into groups based on a threshold. Finally, the modular enrichment analysis computes the enrichment of each gene set not in isolation but rather by incorporating term–term relations into the statistical model. A term is a set of genes which are all involved in the same biological process and are thus functionally related. These term–term relations represent dependencies between gene sets, which can arise, for example, due to shared genes. This approach has the advantage of not requiring arbitrary thresholds to prepare the input genes and is able to incorporate additional biological knowledge into the enrichment computation by imposing a structure on the gene set database. This additional biological knowledge can help maintain high statistical power in large, redundant gene set databases or structure the final visual presentation of enrichment scores (Huang *et al.* 2009).

One of the most basic approaches to compute singular enrichments is to use Fisher’s exact test which is based on the hypergeometric distribution and requires a stratification of the input gene set (Fisher 1922). There have been many extensions to this initial approach, including threshold-free methods such as the popular tool GSEA (Subramanian *et al.* 2005) which does not require an a priori stratification of the input and LRPath which formulates the enrichment computation as a regression (Sartor *et al.* 2009). GSEA has been extended to become more computationally efficient and to be able to approximate small *P*-values more accurately (Korotkevich *et al.* 2016, Lachmann *et al.* 2022).

Various methods have been proposed which follow the modular enrichment approach. topGO (Alexa *et al.* 2006) is tailored to the tree structure of the gene sets provided by the Gene Ontology resource and removes local dependencies between GO terms which leads to better performance. By relying on the topology of a tree, it is not applicable to many other gene set sources. Another approach is to reduce the number of pathways which are included in the enrichment computation by removing redundant terms based on the notion of semantic similarity (Yu *et al.* 2010, Wu *et al.* 2021). RedundancyMiner (Zeeberg *et al.* 2011) transforms the GO database prior to the enrichment computation by de-replicating redundant GO categories and thus tries to reduce the amount of noise introduced by overlapping pathways appearing in the enrichment analysis.

These approaches rely on the directed acyclic graph structure of GO terms and cannot be generalized to other pathway databases. GENECODIS (Carmona-Saez *et al.* 2007) incorporates relations between pathways into the enrichment computation by testing for the enrichment of co-occurring pathways. It can in principle be applied to any pathway database, but it is only available as a web-based tool and can thus not be easily used in automated workflows. The same limitation applies to ProfCom (Antonov *et al.* 2008) which computes the enrichment of unions, intersections, and differences in pathways. In addition, it uses a greedy heuristic which does not guarantee to find an optimal solution for each case. MGSA (Bauer *et al.* 2010) embeds all pathways in a Bayesian network and identifies enriched pathways using probabilistic inference. It does, however, not allow to explicitly model pathway relations.

Finally, tools such as EnrichmentMap (Merico *et al.* 2010), ClueGO (Bindea *et al.* 2009), REVIGO (Supek *et al.* 2011) and GOrilla (Eden *et al.* 2009) compute a singular enrichment score per pathway and subsequently visualize the result as a network of gene set clusters based on gene overlaps. This approach can be applied to any gene set database but loses statistical power by executing the enrichment analysis and term-term relation inclusion in separate steps. GSEA has also been extended to compute the enrichment of functional gene sets instead of individual genes (Han *et al.* 2019). However, it is only available as a web-tool and thus not usable for large-scale, automated analyses. It furthermore suffers from long runtimes and the inability to estimate small *P*-values as it is based on the original GSEA implementation. Other web-based tools which include network-based enrichment methods (Wang *et al.* 2017) can also not be included in automated analyses.

It has also been shown that combining multiple enrichment methods can improve the robustness and interpretability of the results (Alhamdoosh *et al.* 2017). As such approaches ultimately rely on well-performing individual tools, developing novel modular enrichment methods is helpful in this context.

While many methods exist which try to overcome the issue of large redundant pathway databases, none of them, to the best of our knowledge, has accomplished this goal in a simultaneously database-agnostic, flexible, and robust way. By not relying on the hierarchical structure of the Gene Ontology it is possible to create a method which is less restricted and can be used with other pathway databases that are more specialized to the experiment at hand. As there are various approaches to comparing pathways with each other, it is desirable for the enrichment algorithm to not be hard-coded to use a single specific pathway similarity measure but allow different ones based on the needs of the respective research question. The noise inherent to biological experiments leads to measurements of differential gene expression which can deviate from the underlying true differences. Robustness to the level of noise of the input data is thus a crucial property of pathway enrichment methods.

Here, we introduce a novel method called *pareg* for computing pathway enrichments which is based on regularized regression. It follows the ideas of GSEA as it requires no stratification of the input gene list, of MGSA as it incorporates term-term relations in a database-agnostic way, and of LRPath as it makes use of the flexibility of the regression approach. By regressing the differential expression *P*-values of genes on their membership in multiple gene sets while using LASSO and gene set similarity-based regularization terms, we require no prior thresholding and incorporate term-term relations into the enrichment computation. We show in a synthetic benchmark that this model is more robust to noise than competing methods and demonstrate in an application to real data from The Cancer Genome Atlas (TCGA) (Tomczak *et al.* 2015) that it is able to recover known pathway associations as well as suggest novel ones.

## 2 Materials and methods

### 2.1 Overview

The input to *pareg* consists of (i) a list of genes, where each gene is associated with a single *P*-value obtained from a differential expression experiment and (ii) a gene set database where a gene can be part of multiple gene sets simultaneously. *pareg’*s approach is general enough to support any kind of experimental value associated with the input genes. Pathway enrichments are then computed by regressing the differential expression *P*-value vector of input genes on a binary matrix indicating gene membership for each gene set in the input database. The estimated coefficient vector captures the degree of association which gene sets have with *P*-values of differentially expressed genes; it can thus be regarded as an enrichment score. To induce sparsity in the coefficient vector and thus in the selected set of enriched pathways, we use the least absolute shrinkage and selection operator (LASSO) regularization term (Tibshirani 1996). Term-term relations are included in the model using a network fusion penalty (Cheng *et al.* 2014, Dirmeier *et al.* 2018).

### 2.2 Regression approach

We use a regularized multiple linear regression model to estimate gene set enrichment scores. Suppose we want to compute the enrichment of *K* pathways using *N* genes. Each gene *g_i_* is associated with a *P*-value *P_i_* from a differential expression analysis for i=1,…,N. We then define the response vector **Y** to be


(1) $$Y={\left(p_{1},\ldots ,p_{N}\right)}^{T}$$

The binary regressor matrix **X** captures the membership information of each gene *g_i_*, i=1,…,N, in pathway *t_j_*, j=1,…,K,


(2) $$X=\left(\begin{array}{cccc} x_{11} & x_{12} & \cdots & x_{1K}\\ x_{21} & x_{22} & \cdots & x_{2K}\\ \vdots & \vdots & \ddots & \vdots \\ x_{N1} & x_{N2} & \cdots & x_{NK} \end{array}\right)$$

with


(3) $$x_{ij}=\{\begin{array}{cc} 1 & \text{if gene}\;i\;\text{is in pathway}\;j\\ 0 & \text{otherwise} \end{array}$$

In the resulting linear model Y=Xβ, the vector of coefficients $\beta ={\left(\beta_{1},\ldots ,\beta_{K}\right)}^{T}$ is estimated using stochastic gradient descent to minimize the objective function where L(β,ϕ|Y,X) is the likelihood and $G={\left(g_{ij}\right)}_{ij}\in {\left(0,1\right)}^{K\times K}$ a pathway similarity matrix, where *g_ij_* describes the similarity between pathway *i* and *j*.


(4) $$\begin{array}{cc} \hat{\beta }=\underset{\beta ,\phi }{\text{arg min}}( & -\text{log}(L(\beta ,\phi |Y,X))+\lambda \|\beta {\|}_{1}\\ & +\psi \sum_{i=1}^{K}\sum_{j=1}^{K}{\left\|\beta_{i}-\beta_{j}\right\|}_{2}^{2}g_{ij}) \end{array}$$

To model the *P*-values in the response vector, the likelihood is defined using the beta distribution (Ferrari and Cribari-Neto 2004) where p=μϕ and q=(1−μ)ϕ with mean 0<μ<1, precision parameter ϕ>0 and Gamma function Γ(·). The mean is then modeled as g(μ)=Xβ where g(·) is a link function (Cribari-Neto and Zeileis 2010).


(5) $$L(\beta ,\phi |Y,X)=\prod_{i=1}^{N}\left[\frac{\Gamma (p+q)}{\Gamma (p)\Gamma (q)}Y_{i}^{p-1}{\left(1-Y_{i}\right)}^{q-1}\right]$$

The optimal values for the regularization parameters *λ* (LASSO) and *ψ* (network fusion) are determined using cross-validation (Dirmeier *et al.* 2018), which balances the effects of the LASSO and network fusion terms. The former term induces a sparse coefficient vector, i.e. it reduces the number of enriched pathways needed to explain the observed data. The latter term promotes assigning a similar enrichment score to (functionally) similar pathways.

### 2.3 Pathway similarity measures

The goal of adding pathway similarities to the model is to group pathways in the enrichment computation. By doing so, redundant sets of functionally related pathways jointly drive the enrichment signal and reduce the influence of noisy measurements. Due to the flexibility of our model, this can be any similarity measure which can be stored as a real matrix.

As pathways are typically defined as lists of genes, the Jaccard similarity and overlap coefficients are common choices (Merico *et al.* 2010). They group pathways which share many genes together and are thus a good measure of functional relation (Bass *et al.* 2013). The overlap coefficient is particularly suited for pathway collections which feature a hierarchical structure.

In addition, when using the popular Gene Ontology (Gene Ontology Consortium 2004) as a pathway database, semantic similarity measures exist. These measures incorporate the topological structure of the Gene Ontology and are better at inferring functional relations between pathways (Guo *et al.* 2006, Ehsani and Drabløs 2016, Zhao and Wang 2018).

### 2.4 Presentation of enrichment results

The estimated coefficient vector *β* can be ordered descendingly by absolute value such that the most dysregulated and thus interesting pathways appear at the top of the list. A regression coefficient *β_j_* of large absolute value corresponds to a strong dysregulation of pathway *j*.

In addition, we implement a network-based visualization of the enrichment result. Each node in this network corresponds to a pathway, and edges correspond to high pathway similarities. The nodes are colored by the respective enrichment score of each pathway. This allows for the quick identification of functional modules as network clusters.

Finally, the result of *pareg* can be transformed into a format readily understood by the functional enrichment visualization R package enrichplot (Yu 2022). This enables the usage of many plotting functions, such as dot plots, tree plots, and UpSet plots, as well as immediate access to newly implemented ones.

### 2.5 Generation of synthetic data

The goal of the synthetic benchmark is to create a known set of dysregulated pathways which induces a set of differentially expressed genes, apply several enrichment methods (listed below) to this dataset and evaluate how well each method is able to recover the initially dysregulated pathways. Thus, each synthetic dataset consists of a list of genes with associated *P*-values obtained from a simulated differential expression experiment, as well as a respective ground truth set of pathways.

Given an existing term database $D=\{T_{1},\ldots ,T_{K}\}$ consisting of *K* terms $T_{j}=\{g_{1},\ldots ,g_{L_{j}}\}$, each made up of *L_j_* genes *g_i_*, we randomly sample a ground truth set of activated terms $D_{A}\subset D$. In order to model the joint activation of functionally related pathways, we apply a similarity sampling approach. Given a similarity matrix *S* with $0\leq s_{ij}\leq 1$ and similarity factor 0≤ρ≤1 we first uniformly sample a single term *j*. The next term is then drawn according to the probability vector $(1-\rho )U+\rho S_{j}$ where *S_j_* is column *j* of *S* and denotes the similarity of term *j* to all other terms, and *U* is a vector of length $\left|S_{j}\right|$ with values $\frac{1}{\left|S_{j}\right|}$. This procedure is continued by setting *j* to the previously sampled term and repeated until the required number of terms have been sampled. For *ρ* close to 1 this results in similar pathways being sampled, while *ρ* close to 0 leads to a uniformly random sample.

Next, we model synthetic differential expression *P*-values for the *N* genes $\left(g_{1},\ldots ,g_{N}\right)$ by sampling from a Beta distribution whose parameters are determined from a linear combination of a noisy gene-term membership matrix and a term activation vector. This mimics the real-life setting where the dysregulation of a pathway is jointly driven by the dysregulated genes it contains.

In particular, we create the activation vector $\beta_{A}={\left(b_{1},\ldots ,b_{K}\right)}^{T}$ with


(6) $$\begin{array}{cc} b_{k} & ∼\{\begin{array}{cc} -1 & \text{if}\;T_{k}\in D_{A}\\ 0 & \text{otherwise} \end{array} \end{array}$$

That is, we assign a non-zero coefficient to activated pathways. The gene-term membership matrix $X_{A}$ is defined analogously to Equations (2) and (3). To model the effect of noisy measurements, we remove the association between genes and activated terms in $X_{A}$ by setting a fraction of *η* entries to 0. Next, we compute $\mu =g^{-1}\left(X_{A}\beta_{A}\right)$ where $g^{-1}$ is the logistic function and set ϕ=1 to parametrize the Beta distribution. To create the final synthetic dataset $E=(D_{A},\{(g_{i},p_{i}),\ldots ,(g_{N},p_{N})\})$, we sample the differential expression *P*-value *p_i_* for gene *i* from $B(\mu_{i},\phi )$.

We run 20 replicates with 20 activated terms each and use all pathways with sizes between 50 and 500 in the biological process subtree of the Gene Ontology.

### 2.6 Performance evaluation in synthetic benchmark

Due to the strong class imbalance in the experimental setup of pathway enrichments featuring few positives, i.e. dysregulated pathways, compared to the number of negatives, i.e. unaffected pathways, we use precision-recall (PR) curves to evaluate the performance of each pathway enrichment method (Davis and Goadrich 2006, Saito and Rehmsmeier 2015).

A term *T_j_* is classified as a true positive (TP) if it is in *D_A_* and is enriched according to a method and respective threshold. It is classified as a false positive (FP) if it is not a member of *D_A_* but is estimated to be enriched. Analogously, a true negative (TN) is a term which is not in *D_A_* and is not enriched, while a false negative (FN) is a term which is in *D_A_* but is not detected by a method. Precision is then defined as TP/(TP+FP) and recall as TP/(TP+FN). By varying the threshold used to create the classifications, we can then readily create PR curves. To obtain a numeric summary of a method’s performance, we compute the area under the precision-recall curve (AUC).

### 2.7 Real data application

We conduct an exploratory analysis using cancer and normal samples from processed TCGA data available in the Gene Expression Omnibus entry GSE62944 (Rahman *et al.* 2015). We retrieved 113 tumor and matched normal samples for TCGA-BRCA (Breast Invasive Carcinoma). We then use limma (Ritchie *et al.* 2015) to run a differential gene expression analysis to compare tumor and normal samples. The obtained *P*-values and pathways from the biological process subtree of the Gene Ontology are then used as input to *pareg*. We use the Jaccard similarity to create a similarity matrix for all considered pathways. As in the synthetic benchmark, we use all pathways with sizes between 50 and 500 in the biological process subtree of the Gene Ontology.

## 3 Results

First, we demonstrate the effect of the regularization terms used in the objective function and compare the performance of *pareg* to competing methods using a synthetic benchmark study. Second, we conduct an exploratory analysis using a breast cancer dataset from TCGA.

### 3.1 Synthetic benchmark

We compare the performance of *pareg* to other enrichment tools and versions of itself using a synthetic dataset where the ground truth is known. To do so, we select a set of activated terms and generate differential gene expression *P*-values using a linear model.

To demonstrate how the LASSO and network fusion regularization terms contribute toward an improved enrichment result, we compare four versions of *pareg*: (i) *pareg_noterms* which employs an objective function without LASSO and network fusion penalties, (ii) *pareg_lasso* which only uses the LASSO term, (iii) *pareg_network* which only uses the network fusion term, and (iv) *pareg_network_lasso* which uses both penalties and is the method used in the other sections. Cross-validation was used in all applicable cases to determine optimal regularization parameters.

By varying the noise level *η*, we can assess how the two regularization terms contribute toward the model performance (Supplementary Fig. S1). For low noise levels (η=0.25), the sparsity induced by the LASSO penalty yields greater performance improvements over the model without any regularization compared to using only the network fusion penalty. For larger levels of noise (η=0.75), the network structure used in the network fusion penalty is able to boost performance more than only the LASSO term. In both cases, the version of *pareg* which features both regularization terms can make use of each of them to achieve the best performance, demonstrating that both terms are useful and contribute jointly toward good model performance under different circumstances.

**Figure 1.**
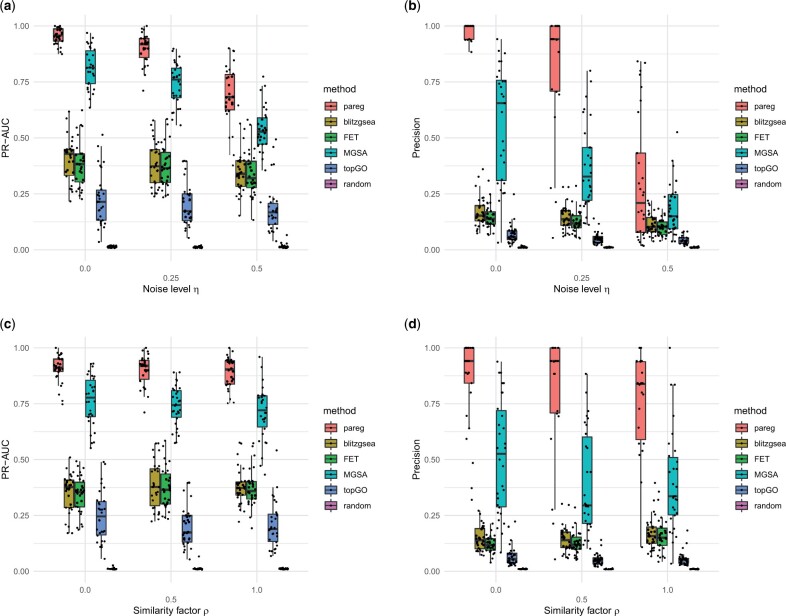
Summary of performance measures calculated for synthetic benchmark. Each point correspond to a single replicate. (a) Boxplots of precision–recall areas under the curve (PR-AUC) for varying noise level η. Individual PR curves are given in Supplementary Figs S3–S5. (b) Boxplots of precision values obtained when setting recall to 0.8 in Supplementary Figs S3–S5 for varying noise level η. (c) Boxplots of precision–recall areas under the curve (PR-AUC) for varying similarity factor ρ. Individual PR curves are given in Supplementary Figs S6–S8. (d) Boxplots of precision values obtained when setting recall to 0.8 in Supplementary Figs S6–S8 for varying similarity factor ρ.

Next, we evaluate the performance of *pareg* by varying the level of noise *η* used when generating synthetic data in order to simulate different real-life situations where noise can arise from measurement errors, as well as the parameter *ρ* which controls the degree of clustering of the enriched terms (Fig. 1).

In addition to *pareg*, we benchmark five other methods. MGSA is a Bayesian approach which embeds pathways in a Bayesian network and explicitly models the activation of sets of pathways (Bauer *et al.* 2010). It constitutes a modular enrichment method of competitive performance to *pareg* which does not depend on a particular pathway database. Fisher’s exact test (FET) is a classical single-term enrichment method which is still commonly used and serves as a simple alternative in the comparison (Fisher 1922). topGO’s elim algorithm incorporates the GO tree structure into the enrichment computation and is a modular enrichment method which relies on using the Gene Ontology (Alexa *et al.* 2006). blitzGSEA provides a computationally performant implementation of GSEA which is based on the pre-rank algorithm and constitutes a popular method for computing enrichments (Lachmann *et al.* 2022). The null model serves as the baseline indicating how random guessing would perform. It assigns a random enrichment *P*-value between 0 and 1 to each pathway.

We observe that *pareg* consistently outperforms all competing methods over a wide range of parameter values (Fig. 1). For varying levels of noise η=0,0.25,0.5 and similarity factor ρ=0,0.5,1, *pareg* achieves the highest mean areas under the precision-recall curve (PR-AUC) in all cases (Fig. 1a and 1c). *pareg* clearly outperforms the singular enrichment methods FET and blitzGSEA, which emphasizes that the proposed method of including term-term relations in the enrichment computation yields an advantage when working with large and redundant pathway databases. Out of all other benchmarked methods, MGSA performs closest to *pareg* indicating that its Bayesian model-based approach which explicitly handles term-term relations in a database-agnostic way is to some extent able to deal with the clustered pathway database. topGO performs slightly worse than FET. It explicitly uses the GO tree structure and performs successive enrichment tests which are individually similar to FET. This approach is not able to appropriately process the clustering structure assumed in the synthetic benchmark which is not based on a tree.

When increasing the noise level *η*, we observe that FET, blitzGSEA and topGO show a smaller decrease in performance than *pareg* and MGSA (Fig. 1a). This is in line with the observation that the precision of FET, blitzGSEA and topGO remains nearly constant when fixing the recall (Fig. 1b). For example, at a recall of 80% *pareg* has a median precision of 94% for η=0.25 while MGSA has a median precision of 37%. FET and topGO have median precision values of 12% and 5% respectively. For *pareg* and MGSA, most PR-AUC is lost for large values of recall where FET and topGO show poor performance even for small *η*. blitzGSEA behaves similarly to FET in those cases. In terms of runtime, due to the optimization routine and cross-validation scheme used in *pareg*, it takes the longest (Supplementary Fig. S2). While the other methods run for less than 2 min, *pareg* takes up to approximately 30 min when run in parallelized mode.

When increasing the similarity factor *ρ*, we see that *pareg* remains at roughly the same PR-AUC (Fig. 1c) and only slightly decreases in precision at a fixed recall level (Fig. 1d), while MGSA shows a stronger decrease in performance. For example, fixing recall to 80% at ρ=0.5 yields a median precision of 94% for *pareg*. MGSA, FET, and topGO have median precision values of 29%, 12%, and 5%, respectively. This indicates that *pareg* is better able to deal with varying levels of clustering in the set of dysregulated pathways. topGO exhibits a slight decline in performance as its tree-based approach is not able to handle the clustering structure induced by the Jaccard similarity measure. As FET and blitzGSEA do not incorporate term-term relations into the enrichment computation, we observe no dependence on *ρ*.

### 3.2 Exploratory analysis of breast cancer samples

**Figure 2.**
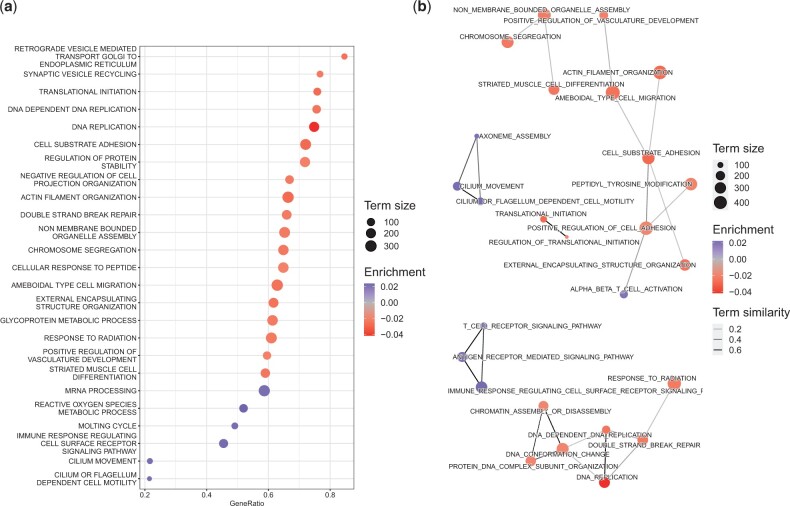
Summary of term enrichment results obtained for TCGA breast cancer samples (normal versus tumor) and the biological process subtree of the Gene Ontology. (a) Top 25 terms ordered by absolute enrichment. The *y*-axis lists the terms while the *x*-axis denotes the fraction of significantly differentially expressed genes (*P*-value < 0.05) over the respective term size. The term size is the number of genes making up the term and also represented as the size of each circle. The color of each circle indicates the enrichment of the respective term where blue corresponds to positive and red to negative enrichment. (b) Non-isolated terms of the 50 terms with largest absolute enrichment. Nodes correspond to terms and edges to Jaccard similarities >0.1. The node color and size has the same meaning as in a. The higher the opacity of an edge the larger the corresponding term similarity.

To investigate the behavior of *pareg* on real data, we use it to run a pathway enrichment analysis on breast cancer (BRCA) samples from TCGA with terms from the Gene Ontology biological process subtree. We order the terms by their absolute enrichment level and list the top 25 results in (Fig. 2a) as well as visualize the top 50 non-isolated results in a network (Fig. 2b).

The largest cluster of the network visualization is made up of 8 nodes and features terms related to cell migration such as ameboidal-type cell migration and actin filament organization. It has been recognized that cancer cells can use amoeboid migration as their preferred migratory strategy (Graziani *et al.* 2021). In particular, it has been shown that treatment via endocrine therapy inhibits this kind of migration in breast cancer. Furthermore, it has been shown that the organization of actin stress fibers promote proliferation of pre-invasive breast cancer cells (Tavares *et al.* 2017). The dysregulation of cell adhesion dynamics has also been investigated in the literature (Maziveyi and Alahari 2017) and is captured by the enrichment of cell-substrate adhesion and positive regulation of cell adhesion terms. In addition, the peptidyl-tyrosine modification term is enriched. Tyrosine acts as a key player in the initiation of proteins at focal adhesion sites. Apart from this, the influence of tyrosine phosphatases on many different cancer types (Motiwala and Jacob 2006) and of tyrosine kinases specifically on breast cancer (Biscardi *et al.* 2000) has been recognized.

The second-largest cluster made up of seven nodes is thematically related to DNA replication and conformational changes. These processes are of high relevance to cancers in general (Jia *et al.* 2017) as well as breast cancer specifically (Ghimire *et al.* 2020). Furthermore, the importance of double-strand break repair has been captured by the enrichment of the corresponding term (Bau *et al.* 2007).

A few smaller clusters remain. One cluster of three nodes contains the terms chromosome segregation, non-membrane-bounded organelle assembly, and striated muscle cell differentiation. The importance of chromosomal stability and the impact of proteins which modulate it have been highlighted for breast cancer (Garcia and Lizcano 2016). Furthermore, it has been observed that breast cancer cells exhibit non-random chromosome segregation (Liu *et al.* 2013). In addition, striated muscle cell differentiation has been linked to the metastatic potential of breast cancer cells (Nikulin *et al.* 2021). Another cluster of three nodes contains the terms cilium movement, cilium or flagellum-dependent cell motility, and axoneme assembly. It has been shown that the expression of cilia is downregulated in various types of cancer, including breast cancer (Higgins *et al.* 2019). It furthermore has impact on the regulation of cancer development (Fabbri *et al.* 2019). The related enrichment of the axoneme assembly terms suggests the importance of the assembly and organization of an axoneme. This constitutes a novel finding and suggests further experimental investigations. The last cluster with three nodes contains the terms T-cell receptor signaling pathway, antigen receptor-mediated signaling pathway, and immune response-regulating cell surface receptor signaling pathway. Both the relevance of the T-cell receptor signaling (Shah *et al.* 2021) and immune response-regulating cell surface receptor signaling term (Rezaei-Tavirani *et al.* 2019) have been recognized. The possibility of investigating the antigen receptor-mediated signaling pathway for a Chimeric antigen receptor T-cell therapy has very recently been considered (Yang *et al.* 2022). Finally, the two-node cluster contains the terms translational initiation and regulation of translational initiation. The regulation of translation via changed expression of the eukaryotic translation initiation factor 3 has been observed to play a positive role in breast cancer progression (Grzmil *et al.* 2010).

In addition to the network clusters, we also detect individually enriched pathways (Fig. 2a). We find the retrograde vesicle-mediated transport, Golgi to endoplasmic reticulum term to be enriched. The potential implications of this apparatus have already been discussed (Spang 2013), but have, to the best of our knowledge, not been linked to breast cancer specifically. The synaptic vesicle recycling term is also enriched. Its potential as a therapeutic target has been recognized (Li and Kavalali 2017), however not in the context of breast cancer. In both cases, our results suggest the novel finding that these pathways may be especially relevant to breast cancer and that further experimental validations in that direction would be interesting.

We also demonstrate the effectiveness of network regularization by comparing the enrichments to results obtained from running *pareg* without the network regularization term (Supplementary Fig. S9) and from FET (Supplementary Fig. S10). In both cases, much fewer clusters are observed, making the biological interpretation more difficult. This indicates that employing the network regularization term is useful for a better understanding of the enrichment results.

## 4 Discussion

We have developed a novel pathway enrichment method called *pareg* which is based on a regularized generalized linear model. It makes use of LASSO and network fusion penalty terms to produce a sparse and coherent list of enriched pathways. The network fusion term incorporates a pathway similarity network which models functional relations between pathways and clusters pathways as part of the enrichment computation in order to handle large and redundant pathway databases.

In a synthetic benchmark, we show that *pareg* is able to outperform single-term enrichment methods such as Fisher’s exact test, a popular tool explicitly including the GO tree in its calculations as well as a model-based approach which embeds pathways in a Bayesian network.

In an exploratory analysis with breast cancer samples, we were able to recover many relevant pathways already known in the literature, as well as suggest novel ones which pose interesting future targets for experimental validation.

We note that *pareg* assumes that a linear combination of gene-pathway memberships is driving the overall pathway dysregulation, an assumption which may reduce the algorithm’s applicability in certain biological environments, such as the interactions between genes in myocardial infarction as measured by mRNA expression profiles (Hartmann *et al.* 2016).

Due to the flexibility of the regression approach, potential future work could go in many directions. Instead of modeling the response variable using a Beta distribution, one may use a beta-uniform mixture which has been suggested for *P*-values (Pounds and Morris 2003). It is also possible to binarize the differential gene expression *P*-values (similarly to how it would have to be done for Fisher’s exact test, a classical enrichment method) into dysregulated and unaffected genes by applying a threshold. The response could then, for example, be modelled using a Bernoulli distribution. This could reduce noisy *P*-value estimation effects but requires the selection of another hyperparameter (the binarization threshold). As the network fusion penalty depends on a general similarity matrix, different measures could be explored. For example, there exist a wide range of different semantic similarity measures which have been used to relate GO terms (Jiang and Conrath 1997, Lin *et al.* 1998, Resnik 1999, Schlicker *et al.* 2006, Wang *et al.* 2007, Zhao and Wang 2018). Alternatively, similarity measures which embed sets of genes in protein-protein interaction networks and compare their localization have been shown to be useful for predicting disease status; they could be another viable choice (Bass *et al.* 2013, Menche *et al.* 2015).

Furthermore, the potential effects of other regularization terms are interesting. Using an Elastic-Net term instead of LASSO or stability selection (Meinshausen and Bühlmann 2010) could improve the sparsity of the coefficient vector. Instead of the network fusion term, regularizations such as hierarchical feature regression (Pfitzinger 2021), regularized k-means clustering (Sun *et al.* 2012) or group LASSO (Yuan and Lin 2006) can be used to incorporate term-term relations and may exhibit more desirable statistical properties, such as stronger robustness to noise, smaller sample size requirements and faster convergence of the optimizer. Due to these regularization terms, it is not immediately possible to compute confidence intervals for each entry of the estimated coefficient vector. The de-biased LASSO approach (Xia *et al.* 2020) can be explored to get a better understanding of the uncertainty involved in the enrichment computation.

Finally, while there have been programming language specific efforts to standardize gene set enrichment benchmarking workflows (Geistlinger *et al.* 2021), no widely accepted consensus has been found. The benchmarking workflow we implement is written in the workflow management system Snakemake (Mölder *et al.* 2021) and thus allows easy integration of additional tools as well as reproducible execution on different back ends. We thus hope that other enrichment tools can use a similar approach to enable comparative benchmarks of new methodologies.

## Supplementary Material

btad522_Supplementary_Data

## Data Availability

The code used to construct the synthetic datasets is available as part of the R/Bioconductor software package *pareg*. The experimental data used in the exploratory analysis is available as GSE62944 on the Gene Expression Omnibus. The pathway database has been obtained from the Gene Ontology resource.

## Code availability

The method *pareg* is freely available as an R package on Bioconductor (https://bioconductor.org/packages/release/bioc/html/pareg.html) as well as on https://github.com/cbg-ethz/pareg. The GitHub repository also contains the Snakemake (Mölder *et al.*, 2021) workflows needed to reproduce all results presented here.

## References

[btad522-B1] Alexa A , Rahnenführer J, Lengauer T et al Improved scoring of functional groups from gene expression data by decorrelating go graph structure. Bioinformatics 2006;22:1600–7. 16606683 10.1093/bioinformatics/btl140

[btad522-B2] Alhamdoosh M , Ng M, Wilson NJ et al Combining multiple tools outperforms individual methods in gene set enrichment analyses. Bioinformatics 2017;33:414–24. 27694195 10.1093/bioinformatics/btw623 PMC5408797

[btad522-B3] Antonov AV , Schmidt T, Wang Y et al Profcom: a web tool for profiling the complex functionality of gene groups identified from high-throughput data. Nucleic Acids Res 2008;36:W347–W351. 18460543 10.1093/nar/gkn239 PMC2447768

[btad522-B4] Bass JIF , Diallo A, Nelson J et al Using networks to measure similarity between genes: association index selection. Nat Methods 2013;10:1169–76. 24296474 10.1038/nmeth.2728 PMC3959882

[btad522-B5] Bau D-T , Mau Y-C, Ding S-L et al DNA double-strand break repair capacity and risk of breast cancer. Carcinogenesis 2007;28:1726–30. 17494053 10.1093/carcin/bgm109

[btad522-B6] Bauer S , Gagneur J, Robinson PN et al Going bayesian: model-based gene set analysis of genome-scale data. Nucleic Acids Res 2010;38:3523–32. 20172960 10.1093/nar/gkq045 PMC2887944

[btad522-B7] Bindea G , Mlecnik B, Hackl H et al Cluego: a cytoscape plug-in to decipher functionally grouped gene ontology and pathway annotation networks. Bioinformatics 2009;25:1091–3. 19237447 10.1093/bioinformatics/btp101 PMC2666812

[btad522-B8] Biscardi JS , Ishizawar RC, Silva CM et al Tyrosine kinase signalling in breast cancer: epidermal growth factor receptor and c-src interactions in breast cancer. Breast Cancer Res 2000;2:203–10. 11250711 10.1186/bcr55 PMC138776

[btad522-B9] Carmona-Saez P , Chagoyen M, Tirado F et al Genecodis: a web-based tool for finding significant concurrent annotations in gene lists. Genome Biol 2007;8:R3–8. 17204154 10.1186/gb-2007-8-1-r3 PMC1839127

[btad522-B10] Cheng W , Zhang X, Guo Z et al Graph-regularized dual lasso for robust eqtl mapping. Bioinformatics 2014;30:i139–i148. 24931977 10.1093/bioinformatics/btu293 PMC4058913

[btad522-B11] Chuang H-Y , Hofree M, Ideker T et al A decade of systems biology. Annu Rev Cell Dev Biol 2010;26:721–44. 20604711 10.1146/annurev-cellbio-100109-104122 PMC3371392

[btad522-B12] Cribari-Neto F , Zeileis A. Beta regression in r. J Stat Soft 2010;34:1–24.

[btad522-B13] Davis J , Goadrich M. The relationship between precision-recall and roc curves. In: *Proceedings of the 23rd International Conference on Machine Learning, Pittsburgh, Pennsylvania USA*, pp. 233–240, June 25–29, 2006, United States: Association for Computing Machinery.

[btad522-B14] Dirmeier S , Fuchs C, Mueller NS et al Netreg: network-regularized linear models for biological association studies. Bioinformatics 2018;34:896–8. 29077797 10.1093/bioinformatics/btx677 PMC6030897

[btad522-B15] Eden E , Navon R, Steinfeld I et al Gorilla: a tool for discovery and visualization of enriched go terms in ranked gene lists. BMC Bioinformatics 2009;10:48–7. 19192299 10.1186/1471-2105-10-48 PMC2644678

[btad522-B16] Ehsani R , Drabløs F. Topoicsim: a new semantic similarity measure based on gene ontology. BMC Bioinformatics 2016;17:296–14. 27473391 10.1186/s12859-016-1160-0 PMC4966780

[btad522-B17] Fabbri L , Bost F, Mazure NM et al Primary cilium in cancer hallmarks. Int J Mol Sci 2019;20:1336. 30884815 10.3390/ijms20061336 PMC6471594

[btad522-B18] Ferrari S , Cribari-Neto F. Beta regression for modelling rates and proportions. J Appl Stat 2004;31:799–815.

[btad522-B19] Fisher RA. On the interpretation of *χ* 2 from contingency tables, and the calculation of p. J Roy Stat Soc 1922;85:87–94.

[btad522-B20] Garcia J , Lizcano F. Kdm4c activity modulates cell proliferation and chromosome segregation in triple-negative breast cancer. Breast Cancer (Auckl) 2016;10:BCBCR.S40182. 10.4137/BCBCR.S40182 PMC5094578 27840577

[btad522-B21] Geistlinger L , Csaba G, Santarelli M et al Toward a gold standard for benchmarking gene set enrichment analysis. Brief Bioinform 2021;22:545–56. 32026945 10.1093/bib/bbz158 PMC7820859

[btad522-B22] Gene Ontology Consortium. The gene ontology (go) database and informatics resource. Nucleic Acids Res 2004;32:D258–61. 14681407 10.1093/nar/gkh036 PMC308770

[btad522-B23] Ghimire H , Garlapati C, Janssen EAM et al Protein conformational changes in breast cancer sera using infrared spectroscopic analysis. Cancers (Basel) 2020;12:1708. 32605072 10.3390/cancers12071708 PMC7407230

[btad522-B24] Graziani V , Rodriguez-Hernandez I, Maiques O et al The amoeboid state as part of the epithelial-to-mesenchymal transition programme. Trends Cell Biol 2022;32:228–42. 34836782 10.1016/j.tcb.2021.10.004

[btad522-B25] Grzmil M , Rzymski T, Milani M et al An oncogenic role of eif3e/int6 in human breast cancer. Oncogene 2010;29:4080–9. 20453879 10.1038/onc.2010.152

[btad522-B26] Guo X , Liu R, Shriver CD et al Assessing semantic similarity measures for the characterization of human regulatory pathways. Bioinformatics 2006;22:967–73. 16492685 10.1093/bioinformatics/btl042

[btad522-B27] Han H , Lee S, Lee I. Ngsea: network-based gene set enrichment analysis for interpreting gene expression phenotypes with functional gene sets. bioRxiv 2019:636498. 10.14348/molcells.2019.0065 PMC6715341 31307154

[btad522-B28] Hanahan D , Weinberg RA. The hallmarks of cancer. Cell 2000;100:57–70. 10647931 10.1016/s0092-8674(00)81683-9

[btad522-B29] Hanahan D , Weinberg RA. Hallmarks of cancer: the next generation. Cell 2011;144:646–74. 21376230 10.1016/j.cell.2011.02.013

[btad522-B30] Hartmann K , Seweryn M, Handelman SK et al Non-linear interactions between candidate genes of myocardial infarction revealed in mRNA expression profiles. BMC Genomics 2016;17:738–14. 27640124 10.1186/s12864-016-3075-6 PMC5027110

[btad522-B31] Higgins M , Obaidi I, McMorrow T et al Primary cilia and their role in cancer. Oncol Lett 2019;17:3041–7. 30867732 10.3892/ol.2019.9942 PMC6396132

[btad522-B32] Huang DW , Sherman BT, Lempicki RA et al Bioinformatics enrichment tools: paths toward the comprehensive functional analysis of large gene lists. Nucleic Acids Res 2009;37:1–13. 19033363 10.1093/nar/gkn923 PMC2615629

[btad522-B33] Jia R , Chai P, Zhang H et al Novel insights into chromosomal conformations in cancer. Mol Cancer 2017;16:173–13. 29149895 10.1186/s12943-017-0741-5 PMC5693495

[btad522-B34] Jiang JJ , Conrath DW. Semantic similarity based on corpus statistics and lexical taxonomy. arXiv, cmp-lg/9709008, 1997, preprint: not peer reviewed.

[btad522-B35] Joshi-Tope G , Gillespie M, Vastrik I et al Reactome: a knowledgebase of biological pathways. Nucleic Acids Res 2005;33:D428–32. 15608231 10.1093/nar/gki072 PMC540026

[btad522-B36] Korotkevich G , Sukhov V, Budin N et al Fast gene set enrichment analysis. *BioRxiv* 2016:060012, preprint: not peer reviewed.

[btad522-B37] Lachmann A , Xie Z, Ma'ayan A et al Blitzgsea: efficient computation of gene set enrichment analysis through gamma distribution approximation. Bioinformatics 2022;38:2356–7. 35143610 10.1093/bioinformatics/btac076 PMC9004650

[btad522-B38] Li YC , Kavalali ET. Synaptic vesicle-recycling machinery components as potential therapeutic targets. Pharmacol Rev 2017;69:141–60. 28265000 10.1124/pr.116.013342 PMC5394918

[btad522-B39] Lin D et al An information-theoretic definition of similarity. In: *International Conference on Machine Learning*, Vol. 98, pp. 296–304, July 24– 27, 1998, United States: Morgan Kaufmann Publishers Inc.

[btad522-B40] Liu W , Jeganathan G, Amiri S et al Asymmetric segregation of template DNA strands in basal-like human breast cancer cell lines. Mol Cancer 2013;12:139–10. 24238140 10.1186/1476-4598-12-139 PMC3866575

[btad522-B72] Lu Xia , Bin Nan, Yi Li.. A revisit to de-biased lasso for generalized linear models. arXiv, arXiv:2006.12778, 2020, preprint: not peer reviewed.

[btad522-B41] Maleki F , Ovens K, Hogan DJ et al Gene set analysis: challenges, opportunities, and future research. Front Genet 2020;11:654. 32695141 10.3389/fgene.2020.00654 PMC7339292

[btad522-B42] Maziveyi M , Alahari SK. Cell matrix adhesions in cancer: the proteins that form the glue. Oncotarget 2017;8:48471–87. 28476046 10.18632/oncotarget.17265 PMC5564663

[btad522-B43] Meinshausen N , Bühlmann P. Stability selection. J Roy Stat Soc 2010;72:417–73.

[btad522-B44] Menche J , Sharma A, Kitsak M et al Uncovering disease-disease relationships through the incomplete interactome. Science 2015;347:1257601. 25700523 10.1126/science.1257601 PMC4435741

[btad522-B45] Merico D , Isserlin R, Stueker O et al Enrichment map: a network-based method for gene-set enrichment visualization and interpretation. PLoS One 2010;5:e13984. 21085593 10.1371/journal.pone.0013984 PMC2981572

[btad522-B46] Mölder F , Jablonski KP, Letcher B et al Sustainable data analysis with snakemake. F1000Res 2021;10:33. 34035898 10.12688/f1000research.29032.1 PMC8114187

[btad522-B47] Motiwala T , Jacob ST. Role of protein tyrosine phosphatases in cancer. Prog Nucl Acid Res Mol Biol 2006;81:297–329. 10.1016/S0079-6603(06)81008-1 PMC3077959 16891175

[btad522-B48] Nikulin S , Zakharova G, Poloznikov A et al Effect of the expression of elovl5 and igfbp6 genes on the metastatic potential of breast cancer cells. Front Genet 2021;12:662843. 34149804 10.3389/fgene.2021.662843 PMC8206645

[btad522-B49] Ogata H , Goto S, Sato K et al Kegg: kyoto encyclopedia of genes and genomes. Nucleic Acids Res 1999;27:29–34. 9847135 10.1093/nar/27.1.29 PMC148090

[btad522-B50] Pfitzinger J. Cluster regularization via a hierarchical feature regression. arXiv, arXiv:2107.04831, 2021.

[btad522-B51] Pounds S , Morris SW. Estimating the occurrence of false positives and false negatives in microarray studies by approximating and partitioning the empirical distribution of p-values. Bioinformatics 2003;19:1236–42. 12835267 10.1093/bioinformatics/btg148

[btad522-B52] Rahman M , Jackson LK, Johnson WE et al Alternative preprocessing of RNA-sequencing data in the cancer genome atlas leads to improved analysis results. Bioinformatics 2015;31:3666–72. 26209429 10.1093/bioinformatics/btv377 PMC4804769

[btad522-B53] Resnik P. Semantic similarity in a taxonomy: an information-based measure and its application to problems of ambiguity in natural language. jair 1999;11:95–130.

[btad522-B54] Rezaei-Tavirani M , Zamanian-Azodi M, Bashash D et al Breast cancer interaction network concept from mostly related components. Galen Med J 2019;8:e1298. 34466490 10.31661/gmj.v8i0.1298 PMC8343932

[btad522-B55] Ritchie ME , Phipson B, Wu D et al Limma powers differential expression analyses for RNA-sequencing and microarray studies. Nucleic Acids Res 2015;43:e47–e47. 25605792 10.1093/nar/gkv007 PMC4402510

[btad522-B56] Saito T , Rehmsmeier M. The precision-recall plot is more informative than the roc plot when evaluating binary classifiers on imbalanced datasets. PLoS One 2015;10:e0118432. 25738806 10.1371/journal.pone.0118432 PMC4349800

[btad522-B57] Sartor MA , Leikauf GD, Medvedovic M et al Lrpath: a logistic regression approach for identifying enriched biological groups in gene expression data. Bioinformatics 2009;25:211–7. 19038984 10.1093/bioinformatics/btn592 PMC2639007

[btad522-B58] Schlicker A , Domingues FS, Rahnenführer J et al A new measure for functional similarity of gene products based on gene ontology. BMC Bioinformatics 2006;7:302–16. 16776819 10.1186/1471-2105-7-302 PMC1559652

[btad522-B59] Shah K , Al-Haidari A, Sun J et al T cell receptor (TCR) signaling in health and disease. Signal Transduct Target Ther 2021;6:412–26. 34897277 10.1038/s41392-021-00823-w PMC8666445

[btad522-B60] Simillion C , Liechti R, Lischer HEL et al Avoiding the pitfalls of gene set enrichment analysis with setrank. BMC Bioinformatics 2017;18:151–14. 28259142 10.1186/s12859-017-1571-6 PMC5336655

[btad522-B61] Spang A. Retrograde traffic from the Golgi to the endoplasmic reticulum. Cold Spring Harb Perspect Biol 2013;5:a013391. 23732476 10.1101/cshperspect.a013391 PMC3660829

[btad522-B62] Steffen M , Petti A, Aach J et al Automated modelling of signal transduction networks. BMC Bioinformatics, 2002;3:34–11. 12413400 10.1186/1471-2105-3-34 PMC137599

[btad522-B63] Subramanian A , Tamayo P, Mootha VK et al Gene set enrichment analysis: a knowledge-based approach for interpreting genome-wide expression profiles. Proc Natl Acad Sci U S A 2005;102:15545–50. 16199517 10.1073/pnas.0506580102 PMC1239896

[btad522-B64] Sun W , Wang J, Fang Y et al Regularized k-means clustering of high-dimensional data and its asymptotic consistency. Electron J Statist 2012;6:148–67.

[btad522-B65] Supek F , Bošnjak M, Škunca N et al Revigo summarizes and visualizes long lists of gene ontology terms. PLoS One 2011;6:e21800. 21789182 10.1371/journal.pone.0021800 PMC3138752

[btad522-B66] Tavares S , Vieira AF, Taubenberger AV et al Actin stress fiber organization promotes cell stiffening and proliferation of pre-invasive breast cancer cells. Nat Commun 2017;8:15237–18. 28508872 10.1038/ncomms15237 PMC5440822

[btad522-B67] Tibshirani R. Regression shrinkage and selection via the lasso. J Roy Stat Soc 1996;58:267–88.

[btad522-B68] Tomczak K , Czerwińska P, Wiznerowicz M et al The cancer genome atlas (tcga): an immeasurable source of knowledge. Contemp Oncol (Pozn) 2015;19:A68–A77. 25691825 10.5114/wo.2014.47136 PMC4322527

[btad522-B69] Wang J , Vasaikar S, Shi Z et al Webgestalt 2017: a more comprehensive, powerful, flexible and interactive gene set enrichment analysis toolkit. Nucleic Acids Res 2017;45:W130–W137. 28472511 10.1093/nar/gkx356 PMC5570149

[btad522-B70] Wang JZ , Du Z, Payattakool R et al A new method to measure the semantic similarity of go terms. Bioinformatics 2007;23:1274–81. 17344234 10.1093/bioinformatics/btm087

[btad522-B71] Wu T , Hu E, Xu S et al Clusterprofiler 4.0: a universal enrichment tool for interpreting omics data. Innovation (Camb) 2021;2:100141. 34557778 10.1016/j.xinn.2021.100141 PMC8454663

[btad522-B73] Yang Y-H , Liu J-W, Lu C et al Car-t cell therapy for breast cancer: from basic research to clinical application. Int J Biol Sci 2022;18:2609–26. 35414783 10.7150/ijbs.70120 PMC8990477

[btad522-B74] Yu G (2023). *enrichplot: Visualization of Functional Enrichment Result*. R package version 1.20.1, Bioconductor, https://yulab-smu.top/biomedical-knowledge-mining-book/.

[btad522-B75] Yu G , Li F, Qin Y et al Gosemsim: an r package for measuring semantic similarity among go terms and gene products. Bioinformatics 2010;26:976–8. 20179076 10.1093/bioinformatics/btq064

[btad522-B76] Yuan M , Lin Y. Model selection and estimation in regression with grouped variables. J Roy Stat Soc 2006;68:49–67.

[btad522-B77] Zeeberg BR , Liu H, Kahn AB et al Redundancyminer: de-replication of redundant go categories in microarray and proteomics analysis. BMC Bioinformatics 2011;12:52–9. 21310028 10.1186/1471-2105-12-52 PMC3223614

[btad522-B78] Zhao C , Wang Z. Gogo: an improved algorithm to measure the semantic similarity between gene ontology terms. Scientific Reports, 2018;8:15107–10. 30305653 10.1038/s41598-018-33219-y PMC6180005

