# Supplementary material for: Coherent pathway enrichment estimation by modeling inter-pathway dependencies using regularized regression
Source: Bioinformatics. 2023 Aug 23;39(8):btad522. doi: 10.1093/bioinformatics/btad522 (PMC10471899; doi:10.1093/bioinformatics/btad522)
Supplement: btad522_Supplementary_Data [file btad522_supplementary_data.pdf]

Supplement: Coherent pathway enrichment estimation by modeling inter-pathway dependencies using regularized regression

Kim Philipp Jablonski[1,2] and Niko Beerenwinkel[1,2,*]

[1]Department of Biosystems Science and Engineering, ETH Zurich, Basel, 4058, Switzerland
[2]SIB Swiss Institute of Bioinformatics, Basel, 4058, Switzerland
[*]To whom correspondence should be addressed

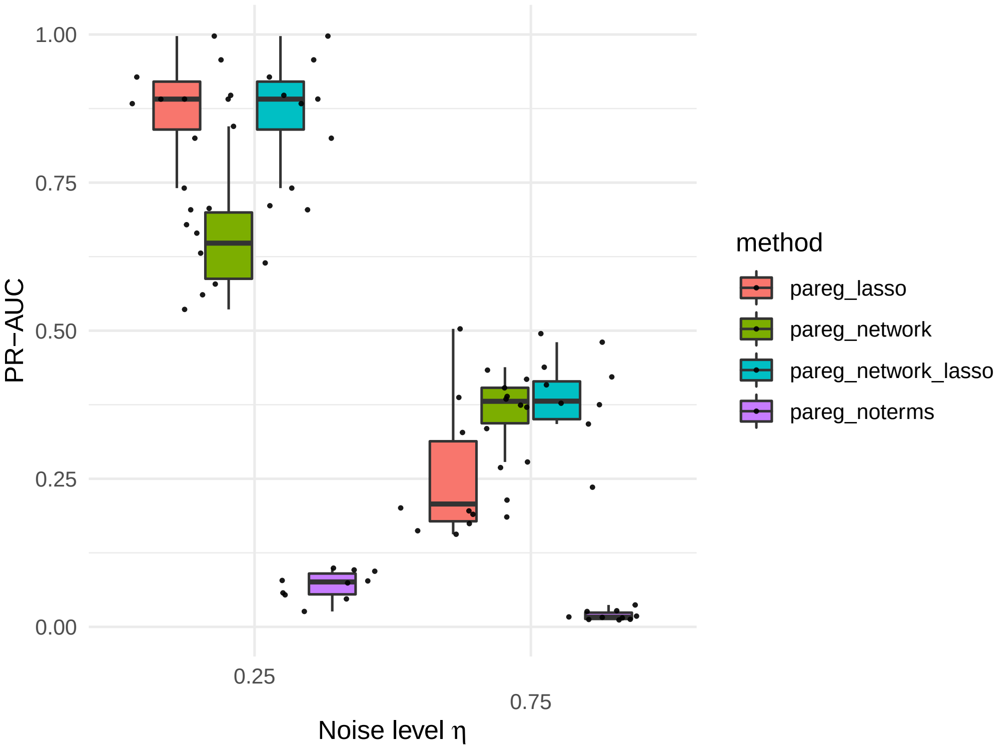

Figure S1: Precision-Recall (PR) areas-under-the-curve (AUC) boxplots for four different versions of *pareg* with differing objective functions ((i) *pareg_noterms* which employs an objective function without LASSO and network fusion penalties, (ii) *pareg_lasso* which only uses the LASSO term, (iii) *pareg_network* which only uses the network fusion term, and (iv) *pareg_network_lasso* which uses both penalties and is the method used in the other sections.) for two noise levels.

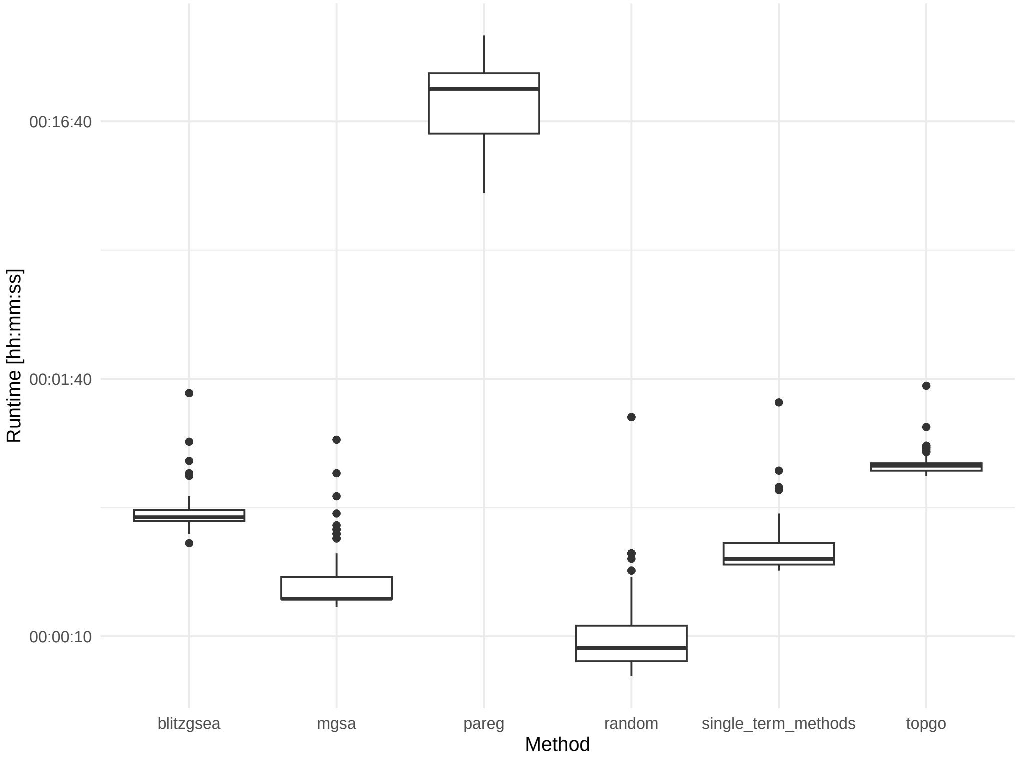

Figure S2: Runtime summary of all methods benchmarked for different levels of noise. Note that the runtime duration includes the time needed to setup the run of each method which is roughly the same between all methods.

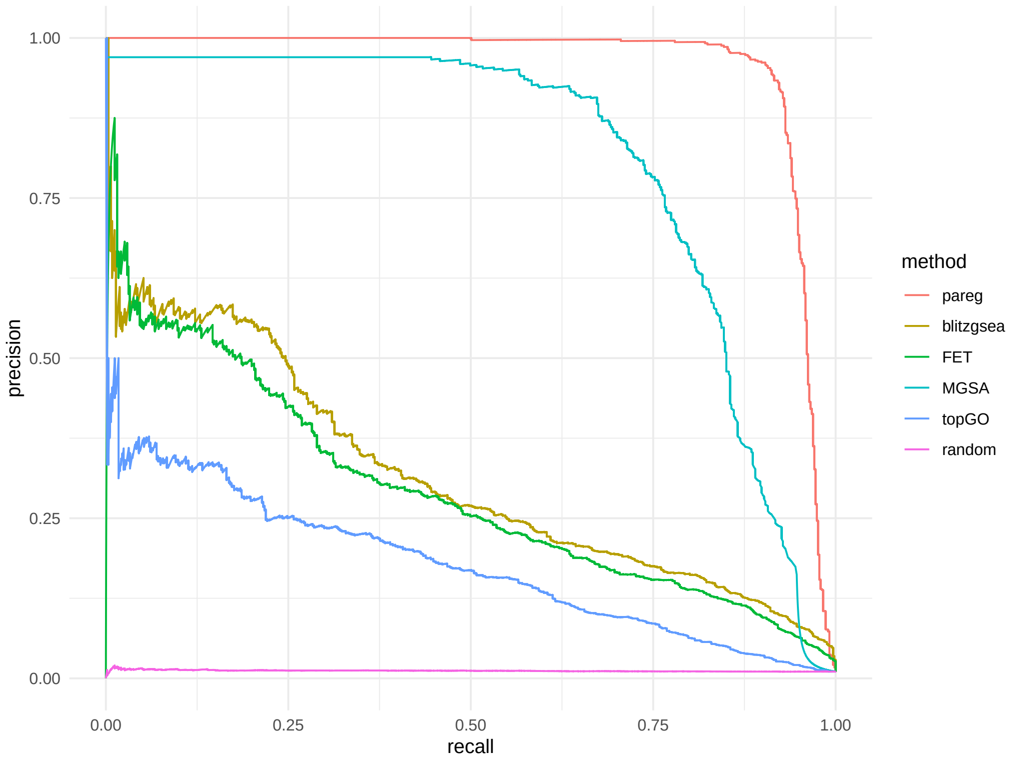

Figure S3: Precision-Recall (PR) curves aggregated over all replicates for noise level $\eta = 0$.

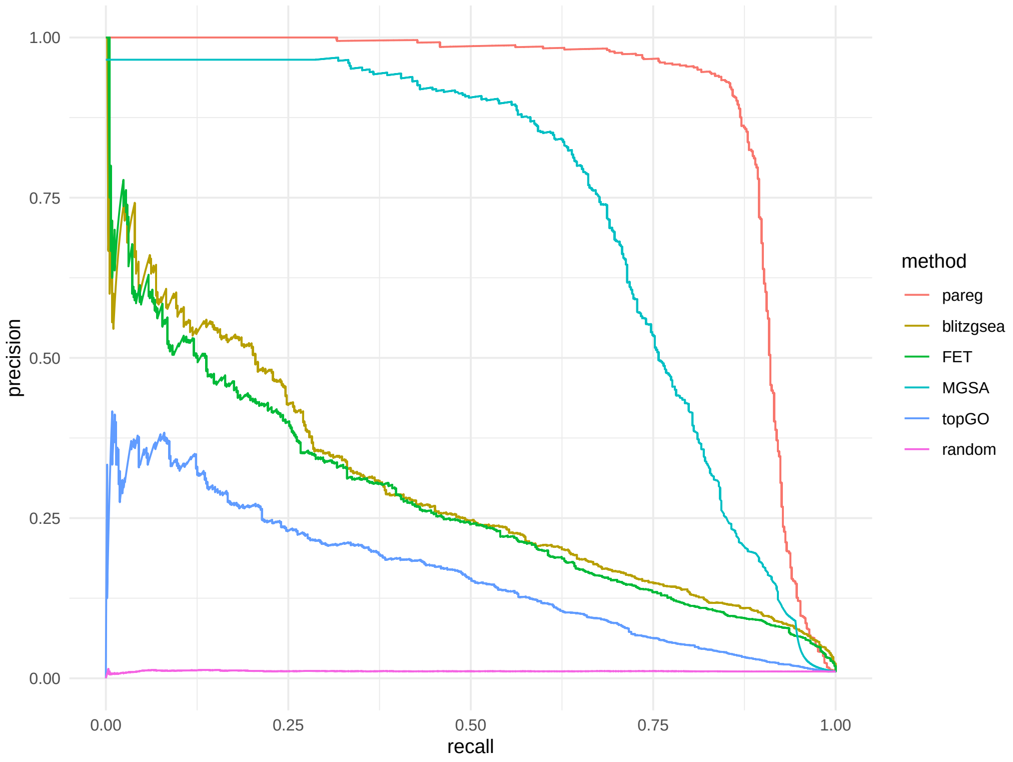

Figure S4: Precision-Recall (PR) curves aggregated over all replicates for noise level $\eta = 0.25$.

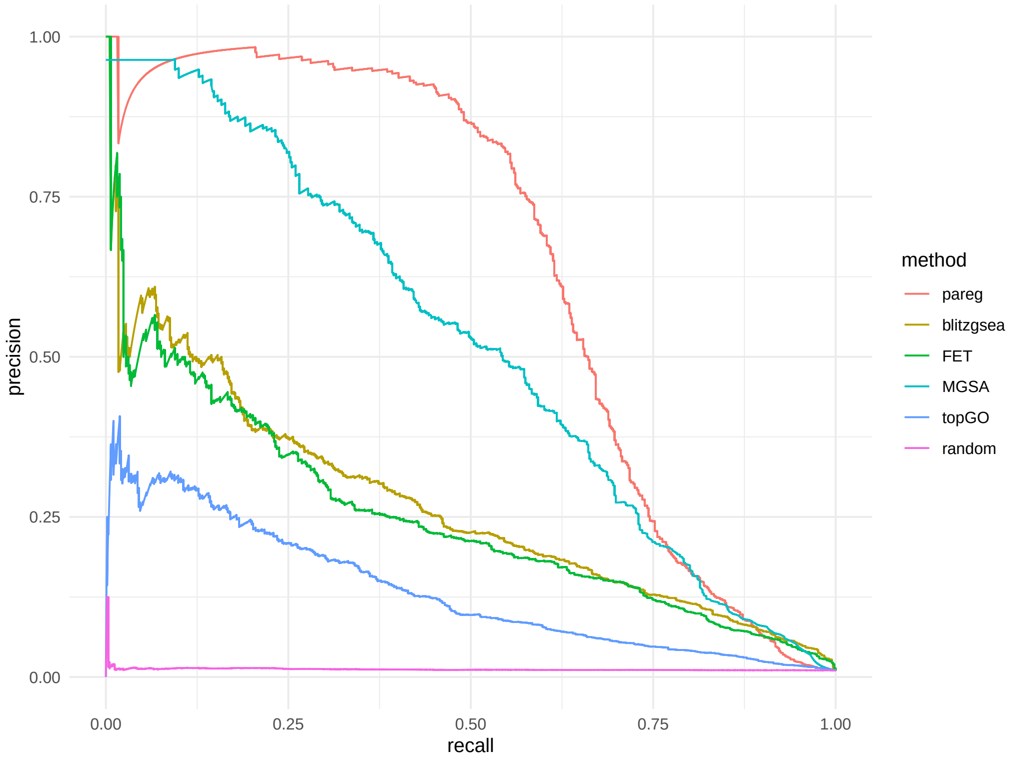

Figure S5: Precision-Recall (PR) curves aggregated over all replicates for noise level $\eta = 0.5$.

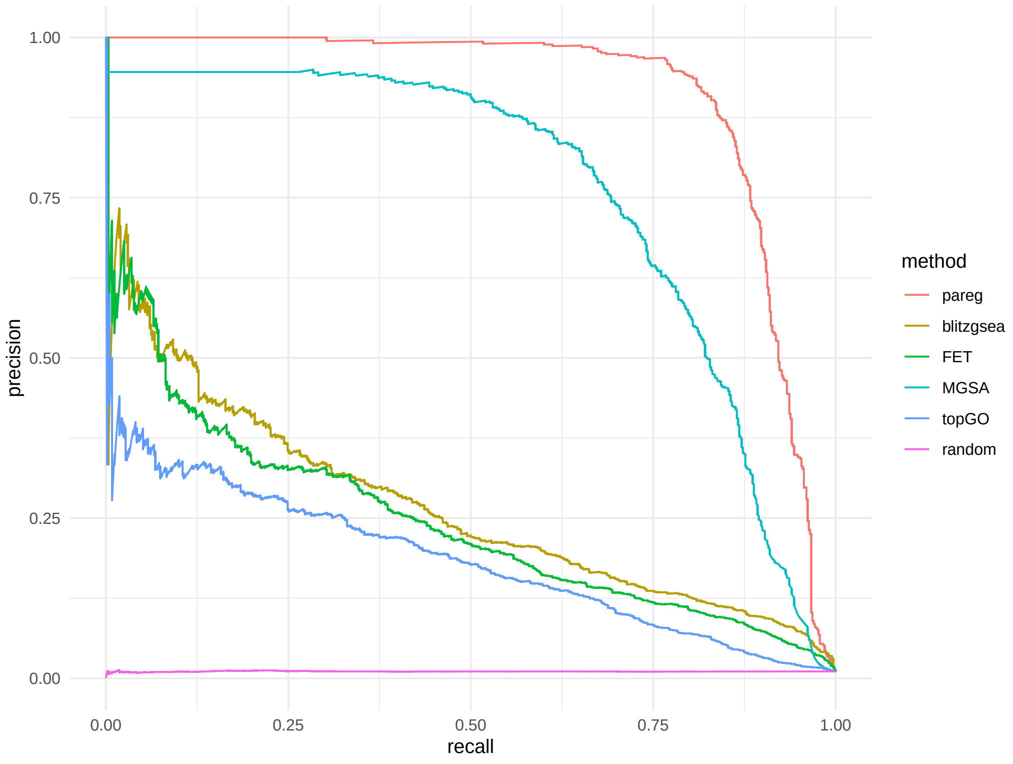

Figure S6: Precision-Recall (PR) curves aggregated over all replicates for similarity factor $\rho = 0$.

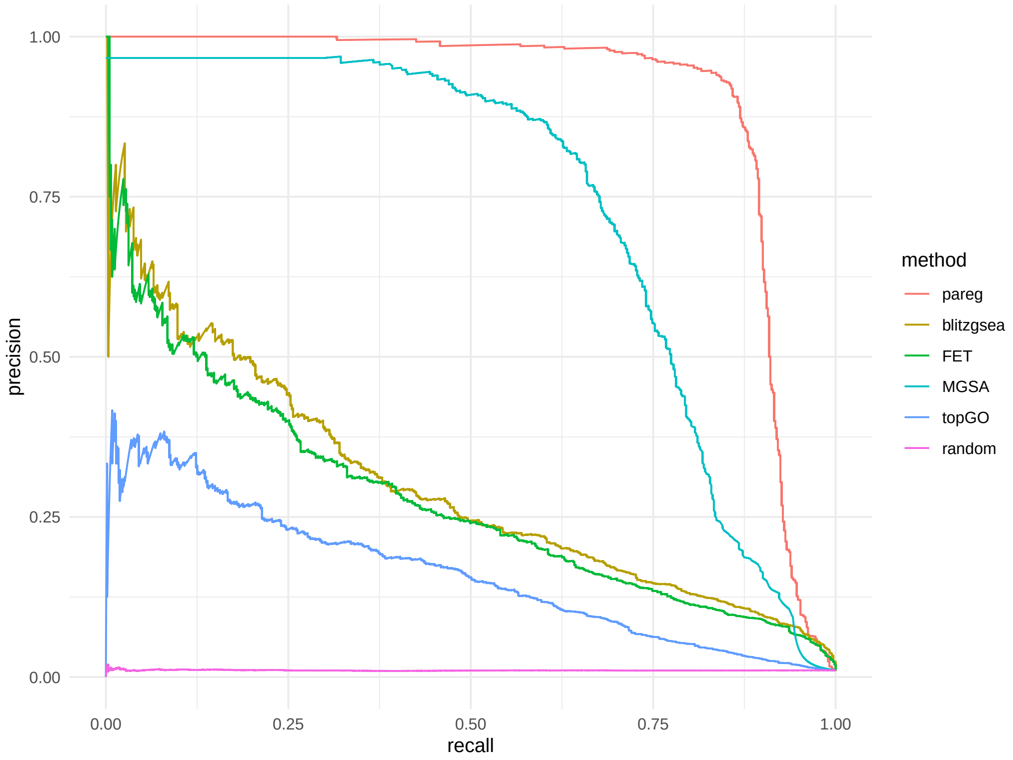

Figure S7: Precision-Recall (PR) curves aggregated over all replicates for similarity factor $\rho = 0.5$.

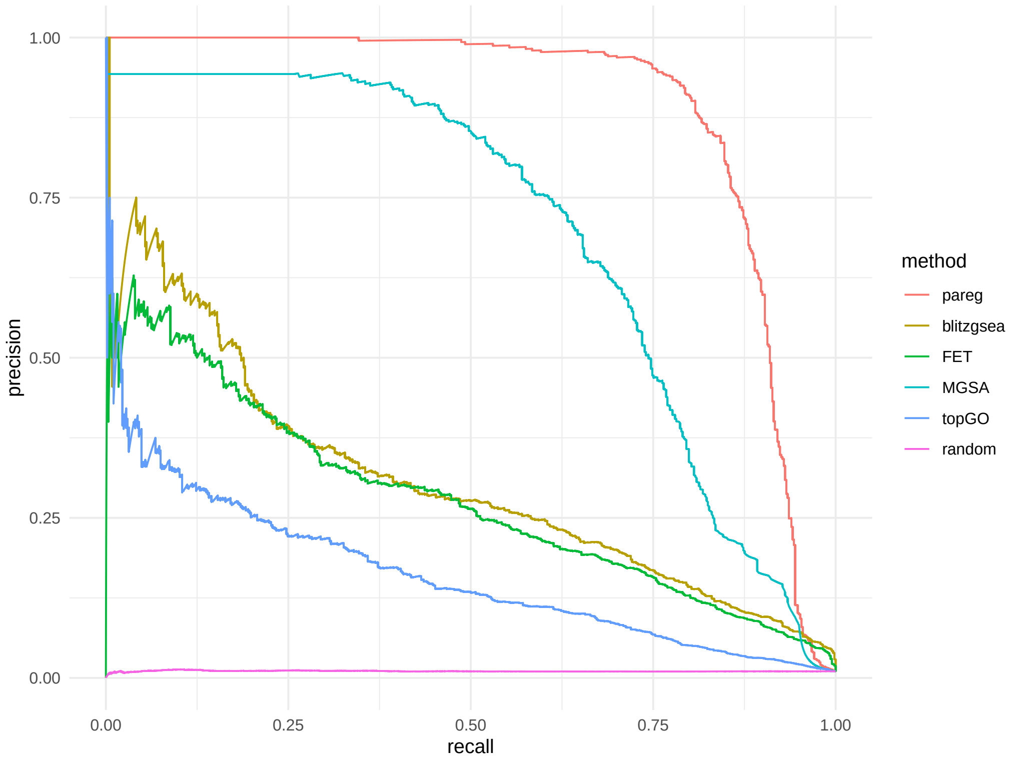

Figure S8: Precision-Recall (PR) curves aggregated over all replicates for similarity factor $\rho = 1$.

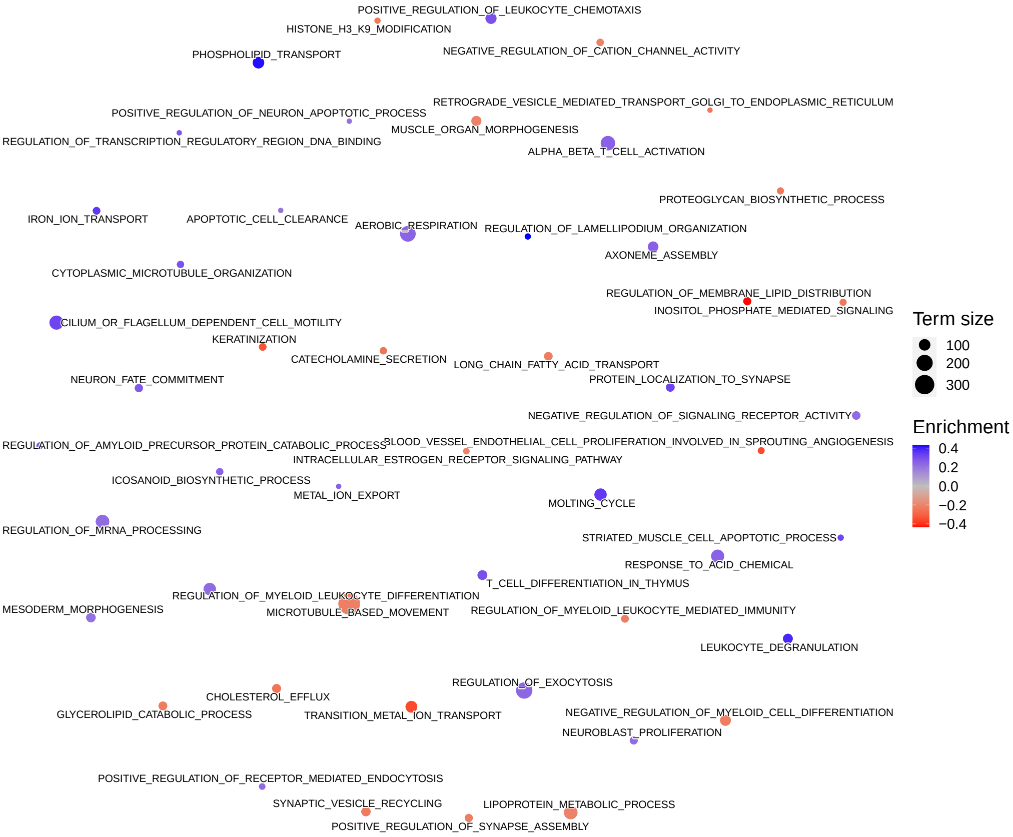

Figure S9: Term network for *pareg* without network regularization with same parameters as in fig. 2b) except for also including isolated terms.

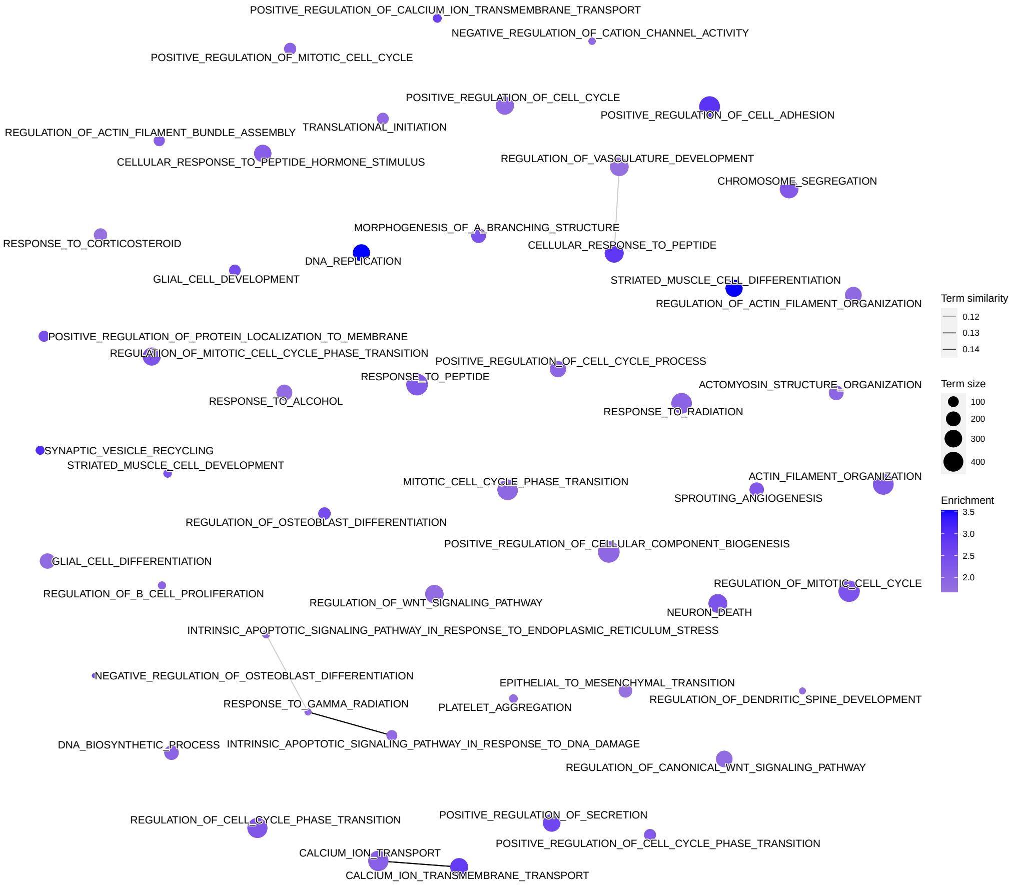

Figure S10: Term network for FET with same parameters as in fig. 2b) except for also including isolated terms. The enrichment score is the negative decadic logarithm of the p-value.
